# Rejection of Quack Advertisements

**Published:** 1845-03

**Authors:** 


*Rejection of Quack Advertisements.*—The following admirable remarks on quackery and quack advertisements are to be found in the No. for November, 1844, of a religious periodical, which is stated by a correspondent to circulate more than 30,000 Nos. monthly, called The Christian Witness:—"We fell into the current, and followed the bad example of pre-existing religious periodicals; but reflection has led us to see our mistake, and we hasten to repair it, assured that we shall give satisfaction to all our readers, who properly estimate the true character of modern quackery, which is one of the vilest and foulest of all foul and vile vocations, and is sustained to an incredible extent by fraud, forgery and falsehood, and fraught with delusion, disease, and death. To publish their nostrums, is to partake of their deeds; to receive their money, is to share their spoils, and aid them in making war upon mankind. No vehicle renders them such assistance in the work of rapine as the *religious magazines,* which, among the thoughtless masses, powerfully and naturally tend to dignify the hateful system, and to sanctify the ruthless imposture. On this point the communications of some of our correspondents are both startling and grievous, and such as show the necessity of religious men and religious magazines cutting all connection with quacks and quackery."—*Lancet.*

